# Enlarging the Toolbox for Allergen Epitope Definition with an Allergen-Type Model Protein

**DOI:** 10.1371/journal.pone.0111691

**Published:** 2014-10-30

**Authors:** Hanna Berkner, Christian Seutter von Loetzen, Maximilian Hartl, Stefanie Randow, Michaela Gubesch, Lothar Vogel, Felix Husslik, Andreas Reuter, Jonas Lidholm, Barbara Ballmer-Weber, Stefan Vieths, Paul Rösch, Dirk Schiller

**Affiliations:** 1 Department of Biopolymers, University of Bayreuth, Bayreuth, Bavaria, Germany; 2 Division of Allergology, Paul-Ehrlich-Institut, Langen, Hesse, Germany; 3 ImmunoDiagnostics Division, Thermo Fisher Scientific Inc., Uppsala, Uppsala län, Sweden; 4 Department of Dermatology, Allergy Unit, University Hospital Zürich, Zürich, Zürich, Switzerland; University of Cambridge, United Kingdom

## Abstract

**Background:**

Birch pollen-allergic subjects produce polyclonal cross-reactive IgE antibodies that mediate pollen-associated food allergies. The major allergen Bet v 1 and its homologs in plant foods bind IgE in their native protein conformation. Information on location, number and clinical relevance of IgE epitopes is limited. We addressed the use of an allergen-related protein model to identify amino acids critical for IgE binding of PR-10 allergens.

**Method:**

Norcoclaurine synthase (NCS) from meadow rue is structurally homologous to Bet v 1 but does not bind Bet v 1-reactive IgE. NCS was used as the template for epitope grafting. NCS variants were tested with sera from 70 birch pollen allergic subjects and with monoclonal antibody BV16 reported to compete with IgE binding to Bet v 1.

**Results:**

We generated an NCS variant (Δ29NCS_N57/I58E/D60N/V63P/D68K_) harboring an IgE epitope of Bet v 1. Bet v 1-type protein folding of the NCS variant was evaluated by ^1^H-^15^N-HSQC NMR spectroscopy. BV16 bound the NCS variant and 71% (50/70 sera) of our study population showed significant IgE binding. We observed IgE and BV16 cross-reactivity to the epitope presented by the NCS variant in a subgroup of Bet v 1-related allergens. Moreover BV16 blocked IgE binding to the NCS variant. Antibody cross-reactivity depended on a defined orientation of amino acids within the Bet v 1-type conformation.

**Conclusion:**

Our system allows the evaluation of patient-specific epitope profiles and will facilitate both the identification of clinically relevant epitopes as biomarkers and the monitoring of therapeutic outcomes to improve diagnosis, prognosis, and therapy of allergies caused by PR-10 proteins.

## Introduction

Millions of patients with allergies to tree pollen are sensitized (produce IgE antibodies) to the major allergen of birch (*Betula verrucosa*) pollen, Bet v 1 [1]. Bet v 1-binding IgE cross-reacts with Bet v 1-homologous proteins from plant foods, leading to allergy to such foods in a majority of birch pollen-allergic subjects [2]. Despite all efforts so far, a complete IgE epitope profile of Bet v 1 or of a Bet v 1-related allergen is still lacking, although knowledge of clinically relevant IgE binding determinants would benefit diagnosis, therapy, and current understanding of the sometimes puzzling clinical phenomena of pollen-related food allergies. Structural information on IgE epitopes of Bet v 1 and Bet v 1-like allergens in foods is limited. To date only IgE epitopes of allergens Art v 1 (pollen), Phl p 2 (grass), and β-lactoglobulin (milk) were determined from X-ray crystallography and NMR spectroscopy of IgE-allergen complexes [3]–[5]. Conformational IgE epitopes of allergens from the PR10-protein family, however, are unknown. Allergen variants carrying substitutions of either individual or multiple residues to either attenuate or induce IgE antibody binding (epitope grafting) identified individual residues crucial for IgE recognition by Bet v 1 [6]–[10] and Bet v 1-type allergens from apple [11], [12], cherry [13], [14], and celeriac [15]. In addition, monoclonal antibodies have been used to localize potential IgE epitopes of Bet v 1 and related allergens [10], [16]–[19]. The only structural information on an epitope of Bet v 1 to date was obtained from X-ray crystallography of a complex between the monoclonal Bet v 1-specific mouse IgG BV16 and the major isoform of Bet v 1, Bet v 1 a [20]. Binding of BV16 to Bet v 1 reduces serum IgE interactions, indicating competition of IgE and monoclonal IgG for an overlapping binding site of Bet v 1 [21], [22].

IgE-allergen interactions are usually analyzed with polyclonal serum IgE, making it difficult to differentiate individual IgE interaction sites. Engineering of recombinant allergen variants often changes the native protein conformation which is essential for IgE interaction. Thus we suggest to use a non-IgE binding protein with allergen-like conformation as a scaffold that can be modulated on the individual amino acid level to stepwise induce IgE recognition capabilities. For this purpose we recently cloned, expressed, purified, and characterized the enzyme norcoclaurine synthase (NCS) from the meadow rue *Thalictrum flavum* as a recombinant protein variant Δ29NCS [23], [24]. As Bet v 1, NCS is a member of the pathogenesis-related protein family (PR-10) sharing the typical Bet v 1 protein fold [25], but has no known allergenic properties. NCS is thus an ideal protein model candidate to study epitopes of PR-10 allergens.

Here we aimed at establishing a recombinant model protein system to specifically analyze epitopes of PR-10 allergens. For this purpose we used the truncated variant Δ29NCS. To study the impact of individual amino acids in IgE binding within NCS we generated variants of Δ29NCS, analyzed their IgE antibody binding with sera of birch pollen allergic subjects and determined the cross-reactivity of suspected IgE epitopes grafted onto NCS.

## Methods

### Patients

Sixty-nine patients with a convincing history of pollinosis to early flowering tree pollen and specific IgE levels>0.35 kU_A_/L to birch pollen measured by ImmunoCAP (Thermo Fisher Scientific, Uppsala, Sweden) were included as serum donors. Patients were recruited at the Allergy Unit, Department of Dermatology, University Hospital Zürich, Switzerland, at the Hospital Borkum Riff, Borkum, Germany, and at the Paul-Ehrlich-Institut, Langen, Germany. Study participants provided written informed consent. Ethics approval by the local ethics committee ‘Kantonale Ethikkomission Zürich, Switzerland’ of the University Hospital in Zürich, Switzerland and the local ethics committee ‘Ethik-Kommission, Fachbereich Medizin der Johann Wolfgang Goethe-Universität, Frankfurt am Main, Germany’ of the University Hospital in Frankfurt included consent form and consent procedure. Sera 52–69 were published elsewhere [26]. Serum 70 was purchased from DLab Diagnose GmbH, Hamburg, Germany. Serum from one non-allergic subject was used as negative control for the specific IgE measurements. The positive serum pool (IgE>0.35 kU_A_/L against Δ29NCS_5x) comprised sera 11, 14, 20–23, 32, 42, 47, and 70. The negative serum pool (IgE<0.35 kU_A_/L against Δ29NCS_5x) comprised sera 4, 12, 18, 29–31, 33, 40, 43, and 46.

### Determination of specific IgE

Specific IgE levels to recombinant proteins were determined by ImmunoCAP in Phadia 100 and 250 instruments (Thermo Fisher Scientific, Uppsala, Sweden) according to the manufacturer's instructions. In the case of Bet v 1, Api g 1.01 and Cor a 1.04, commercial ImmunoCAP tests were used, whereas for Pru av 1, Dau c 1.01, NCS and its variants, experimental ImmunoCAPs were manufactured by coupling the recombinant allergens individually to the solid phase, as described elsewhere [27], [28].

### Cloning, expression and purification of Δ29NCS, Bet v 1 variants, and Bet v 1-related allergens

Mutations leading to the amino acid exchanges/insertion in Δ29NCS_N57/I58E/D60N/V63P_, Δ29NCS_N57/I58E/D60N/V63P/D68K_, and Bet v 1_N43A/E45S/N47A/K55A_ were sequentially introduced into pET29b-Δ29NCS [23] and pET15b-Bet v 1, respectively, with the QuickChange kit from Stratagene (Heidelberg, Germany). All protein variants were expressed and purified via their His_6_-tag as described [23] with minor changes. To yield higher amounts of soluble protein, the synthesis of the protein variants was induced by the addition of 1 mM IPTG at 25°C followed by incubation overnight. A synthetic gene encoding soy allergen Gly m 4 was cloned into pET15b and purified as described above for Bet v 1. The following Bet v 1-related allergens were expressed and purified as described: Pru av 1 (cherry) [29], Dau c 1 (carrot) [26], and Cor a 1.04 (hazelnut) [30]. rApi g 1.01 (celeriac) was purchased from Biomay, Vienna, Austria.

### Circular dichroism

Far UV circular dichroism (CD) spectra of the Δ29NCS variants were acquired at 293 K using a Jasco J-810 spectropolarimeter (Japan Spectroscopic, Gross-Umstadt, Germany) at a band width of 1 nm and a sensitivity of 100 mdeg in a 0.2 cm cell. All proteins were analyzed at a concentration of 2.5 µM in 5 mM sodium phosphate, pH 7.0. Each measurement comprised the average of 10 repeated scans between 260 and 190 nm.

### NMR spectroscopy

NMR samples were prepared by dissolving lyophilized ^15^N-labeled protein in 20 mM sodium phosphate, pH 7.0, 1 mM DTT, 0.04% sodium azide and 10% D_2_O. ^1^H-^15^N-HSQC spectra were recorded on a Bruker Avance 700 MHz spectrometer at 298 K. NMR data were processed using in-house software and were visualized with NMR view [31].

### Modelling of NCS variants

Structure predictions of Δ29NCS_N57/I58E/D60N/V63P_, Δ29NCS_N57/I58E/D60N/V63P/D68K_, and Bet v 1_N43I/E45S/N47D/K55A_ were performed by using the Phyre server [32]. The calculated models were based on the structures of wild type NCS [25] (pdb 2VNE) and Bet v 1a [33] (pdb 1BV1), respectively, with a confidence of 100% and a sequence coverage of at least 85%.

### Binding of monoclonal antibody BV16 to Bet v 1 and related allergens

Nunc Maxisorp plates (Fisher Scientific, Schwerte, Germany) were coated overnight at room temperature with 250 ng/100 µl proteins (Δ29NCS_5x; Δ29NCS; Dau c 1; Pru av 1; Bet v 1; Gly m 4; Cor a 1), with 50 ng/100 µl Api g 1, and with 25 ng/100 µl Bet v 1 and Bet v 1_4x in phosphate-buffered saline (PBS). After blocking with PBS containing 2% BSA a dilution series of the murine monoclonal anti-Bet v 1 antibody BV16 [34] was added for 1 h at room temperature in PBS containing 0.05% Tween 20 and 0.1% BSA. Allergen-specific IgG was detected with horseradish peroxidase-conjugated goat anti-mouse IgG antibody (A3673, SigmaAldrich, Taufkirchen, Germany) diluted 1∶3000 in PBS containing 0.05% Tween 20 and 0.1% BSA as described for the IgE ELISA below.

### Inhibition IgE ELISA

For IgE-ELISA inhibition experiments, Nunc Maxisorp plates (Fisher Scientific, Schwerte, Germany) were coated overnight at room temperature with 300 ng/100 µl Δ29NCS_5x with 10 mM potassium phosphate-buffered saline (PBS). After blocking with PBS containing 2% BSA, plates were incubated with the human serum pool (dilution 1∶10) and increasing concentrations (3*10^−5^–3 µg/well) of recombinant inhibitors (Bet v 1, Bet v 1_4x, Dau c 1.01, Pru av 1, Gly m 4, Cor a 1.04, and Api g 1.01, respectively) for 3 hrs at room temperature with PBS containing 0.05% Tween 20 and 0.1% BSA. Allergen-specific IgE was detected with horseradish peroxidase-conjugated mouse anti-human IgE antibody (Clone B3102E8, Southern Biotech via Biozol, Eching, Germany) diluted 1∶1000 with PBS containing 0.05% Tween 20 and 0.1% BSA. The substrate for horseradish peroxidase was 3,3′,5,5′-tetramethylbenzidine (Roth, Karlsruhe, Germany) and the reaction was stopped by addition of 25% H_2_SO_4_. The absorbance was measured at 450 nm.

For the IgE-ELISA inhibition experiment with monoclonal antibody, ascites fluid of Bet v 1-specific antibody BV16 [34] (diluted: 1∶1000; 1∶10000, and 1∶100000 in PBS containing 0.05% Tween and 0.1% BSA) was pre-incubated with 9 ng/well Bet v 1 as inhibitor for 2 hrs at room temperature. As a negative control, dilution buffer alone was preincubated with Bet v 1. After blocking with PBS containing 2% BSA, rBet v 1 and serum pool IgE diluted 1∶10 (final concentration) were incubated on the plate for 3 hrs at room temperature. Human IgE was detected as described below.

### Indirect ELISA for IgE binding to Bet v 1 variants

For IgE-ELISA experiments, Nunc Maxisorp plates (Nunc via Fisher Scientific, Schwerte, Germany) were coated overnight at room temperature with 25 ng/100 µl recombinant Bet v 1 and Bet v 1_4x, respectively with phosphate-buffered saline (PBS). Blocking and IgE detection was done as described for IgE ELISA above.

### SDS-PAGE and immunoblot analysis

SDS-PAGE was performed with 15% separating gels and 5% stacking gels using a discontinuous buffer system [35]. For immunoblot analysis, 0.5 µg/cm of recombinant protein Δ29NCS_5x was transferred onto 0.2 µm nitrocellulose membranes by semi-dry blotting at 0.8 mA/cm^2^ for 1 h [36]. After blocking with Tris-buffered saline (TBS) containing 0.3% Tween 20 blots were cut into strips and incubated overnight at room temperature with 5 µl of human serum pool with or without increasing amounts of recombinant allergens as inhibitors (Δ29NCS_5x and Bet v 1, respectively) with TBS containing 0.05% Tween 20 (TBST 0.05%) and 0.1% BSA (TBST 0.05%–0.1% BSA). After incubation for 1 h with horseradish peroxidase labelled mouse anti-human IgE antibody (Clone B3102E8, Southern biotech via Biozol, Eching, Germany), diluted 1∶100000 with TBST 0.05%–0.1% BSA, IgE-binding proteins were visualized by chemiluminescence (LumiGLO Reserve diluted 1∶3, KPL via Medac, Wedel, Germany).

### Mediator release from humanized Rat Basophil Leukaemia (RBL) cells

The mediator release assay followed an established protocol [37]. Briefly, RBL cells expressing the α-chain of human FcεRI were sensitized overnight with the positive serum pool of human sera (diluted 1∶20). After washing, cells were stimulated with serial dilutions of allergens. Degranulation was quantified by photometric measurement of β-hexosaminidase activity in the culture supernatants and was expressed as percent of the total cellular β-hexosaminidase content obtained by lysing the cells with Triton X-100 (Sigma-Aldrich, Steinheim, Germany) after correction for spontaneous release (sensitized cells without allergen).

### MS analysis of recombinant protein variants

The identity of recombinant proteins was confirmed by liquid chromatography mass spectrometry (LC-MS). Recombinant proteins were excised from Coomassie stained SDS-PAGE gels and analyzed as described elsewhere [38]. Differing from this, peptides were eluted with 25 mM NH_4_HCO_3_; 10% aceto nitrile (ACN) and the digestion was stopped by adding 5% formic acid. The peptides were analyzed by using a nano-ultra performance LC system coupled to a nano-ESI- MS (nano Acquity UPLC nanoESI Synapt-MS, Waters, Milford, US) with a 5 µm symmetry 180 µm×20 mm c18 pre-column and a 1.7 µm BEH 130 100 µm×100 mm c18 separation column. After 3 minutes of trapping (99% water at 5 µl/minute), a 30 minutes gradient (3–40% ACN at 500 nl/minute) was applied to separate peptides. MS was operated in V mode, acquiring MSE data and applying standard parameters. Data analysis was performed with protein lynx global server version 2.4 (Waters), searching an in house database consisting of the Uniprot database (as of May 2011, restricted to reviewed entries of eukaryotic organisms) and the amino acid sequences of the recombinant variants Δ29NCS, Δ29NCS_4x and Δ29NCS_5x. The identification of a protein was accepted at a false positive rate of less than 4%; peptide mass accuracy was 9 parts per million (ppm) or better.

### Miscellaneous

Amino acid sequence alignments were carried out with ClustalO [39]. 3D protein models were analyzed and illustrated using PyMol [40].

## Results

### Δ29NCS variants have Bet v 1-type tertiary structure

**Figure 1:**
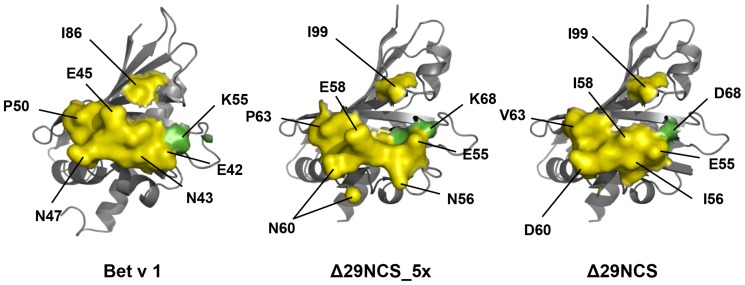
Grafting of an epitope onto Δ29NCS. Left panel: 13 of the 16 amino acids comprising the Bet v 1 epitope (Bet v 1_E42/N43/I44/E45/G46/N47/G48/G49/P50/G51/T52/R70/D72/H76/I86/K97_) of mouse monoclonal IgG antibody BV16 (yellow). Six amino acids of the epitope are labeled. Lys55 of Bet v 1 located adjacent to the epitope is highlighted (green). Middle panel: the corresponding 13 residues of the Bet v 1 epitope for BV16 and a lysine corresponding to K55 of Bet v 1 have been grafted onto Δ29NCS to generate Δ29NCS_4x (Δ29NCS_ N57/I58E/D60N/V63P_) (not shown) and Δ29NCS_5x (Δ29NCS_ N57/I58E/D60N/V63P/D68K_). Right panel: corresponding surface area of Δ29NCS. Protein models were based on the structures of NCS (pdb 2VNE) and Bet v 1a (pdb 1BV1), and modelled with a confidence of 100% and a sequence coverage of at least 85%.

**Figure 2:**
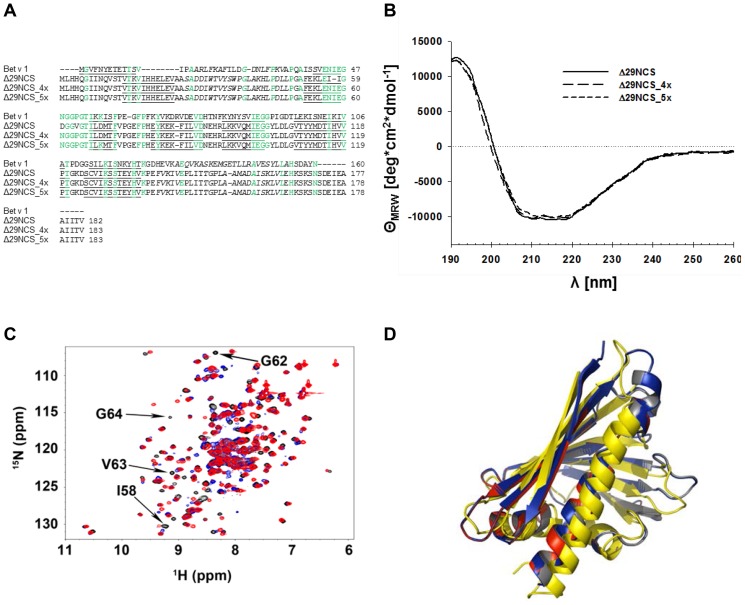
Δ29NCS, Δ29NCS_4x, and Δ29NCS_5x structures are virtually identical to the Bet v 1 structure. (A) Structural sequence alignment. β-strands (underlined), helices (italic), and amino acid identities (green) to Bet v 1 (pdb: 1BV1) are 23.8% (Δ29NCS), 26.8% (Δ29NCS_4x), and 27.5% (Δ29NCS_5x), respectively. (B) Circular dichroism of Δ29NCS variants. (C) Overlay of three ^1^H-^15^N-HSQC spectra of Δ29NCS variants. Black arrows, signals numbered and named according to Δ29NCS. Black: Δ29NCS, blue: Δ29NCS_4x, red: Δ29NCS_5x. The arrows highlight signals of selected amino acids that are expected to differ between the three Δ29NCS variants. Labeling is according to Δ29NCS scheme. (D) Overlay of secondary structure topologies of Δ29NCS (grey), Δ29NCS_4x (blue), Δ29NCS_5x (red), and Bet v 1 (yellow).

IgE antibody binding to PR-10 allergens depends on the presence of a native protein fold of the allergen. The crystal structure of recombinant norcoclaurine synthase (NCS, pdb 2VNE) revealed a conformation identical to that of Bet v 1, even though the sequence identity of these proteins is only 25% [25]. We recently optimized purification of a properly folded N-terminally truncated variant of NCS (Δ29NCS), which comprises the complete structurally corresponding amino acid sequence of Bet v 1 [23]. We generated two Δ29NCS variants harboring single amino acids of a Bet v 1 epitope for IgG that competes with IgE [20], [22] (Figures 1 and 2A). In addition, Asp68 of NCS was exchanged for Lysine, a corresponding amino acid of Bet v 1 involved in IgE binding [22], [41] and located directly adjacent to the epitope. Initially we tested whether the rΔ29NCS variants have the typical Bet v 1 conformation using circular dichroism (CD) and nuclear magnetic resonance (NMR) spectroscopy (Figure 2B, 2C and Figure S1). Both the CD and ^1^H^15^N-HSQC NMR spectra of Δ29NCS_N57/I58E/D60N/V63P_ (Δ29NCS_4x) and Δ29NCS_N57/I58E/D60N/V63P/D68K_ (Δ29NCS_5x) indicated that the amino acid substitutions in the NCS variants did not change the proteins' secondary or tertiary structures and confirmed that the native protein conformation is maintained in the variants (Figure 2D).

### Δ29NCS variants selectively bind IgE from sera of birch pollen allergic subjects

To assess potential cross-reactive IgE binding to rΔ29NCS variants, 70 sera of birch pollen allergic subjects were analyzed for IgE binding to a range of PR-10 proteins using ImmunoCAP tests (Table 1). All sera had sIgE to Bet v 1 (from 0.69 to>100 kU_A_/L) and a majority to the PR-10 food allergens Pru av 1.01 (cherry, <0.35 to 58.2 kU_A_/L), Cor a 1.04 (hazelnut, <0.35 to>100 kU_A_/L), Dau c 1.01 (carrot, <0.35 to 98.5 kU_A_/L), and Api g 1.01 (celeriac, <0.35 to 43.1 kU_A_/L), respectively. In contrast, most of the sera (66/70, 94%) tested negative (<0.35 kU_A_/L) to Δ29NCS, whereas 25/70 (36%) showed significant IgE binding (0.35 to 3.34 kU_A_/L, p <0.0001) to Δ29NCS_4x and 50/70 (71%) to Δ29NCS_5x (0.35 to 17.4 kU_A_/L, p<0.0001). Thus, gradual substitution of certain amino acids of the low-IgE binding protein Δ29NCS with those of Bet v 1 at the corresponding positions induced significant binding of serum IgE from subjects with birch pollinosis.

**Table 1:** Human sera used in this study.

Patient	Serological data: specific IgE (kU_A_/L) and CAP classes (0–6)															
No.	rBet v 1		rPru av 1		rCor a 1.04		Dau c 1.01		rApi g 1.01		Δ29NCS		Δ29NCS_4x _N57/I58E/D60N/V63P_		Δ29NCS_5x _N57/I58E/D60N/V63P/D68K_	
1	73.4	5	32.20	4	78.8	5	5.05	3	5.74	3	0.75	2	3.34	2	11.3	3
2	11.1	3	2.47	2	6.8	3	<0.35	0	<0.35	0	<0.35	0	0.39	1	1.38	2
3	43.3	4	16.1	3	38.3	4	14.1	3	11.8	3	<0.35	0	<0.35	0	2.89	2
4	79.4	5	22.4	4	>100	6	3.36	2	5.95	3	<0.35	0	<0.35	0	<0.35	0
5	48.6	4	12.1	3	57.8	5	6.54	3	6.84	3	<0.35	0	0.99	2	1.75	2
6	89.9	5	34.6	4	>100	6	1.64	2	2.00	2	<0.35	0	<0.35	0	13.8	3
7	69.6	5	18.0	4	55.2	5	9.55	3	10.7	3	<0.35	0	1.74	2	7.34	3
8	>100	6	51.2	5	61.7	5	28.1	4	26.9	4	0.45	1	0.93	2	7.34	3
9	>100	6	41.7	4	71.6	5	45.3	4	43.1	4	<0.35	0	<0.35	0	4.42	3
10	68.4	5	18.7	4	67.3	5	<0.35	0	0.74	2	<0.35	0	<0.35	0	17.4	4
11	>100	6	13.1	3	92.4	5	4.06	3	1.66	2	<0.35	0	<0.35	0	5.15	3
12	5.61	3	1.86	2	4.88	3	<0.35	0	0.39	1	<0.35	0	<0.35	0	<0.35	0
13	54.3	5	2.79	2	25.9	4	1.22	2	1.59	2	<0.35	0	<0.35	0	0.55	1
14	24.2	4	7.71	3	29.3	4	<0.35	0	1.28	2	<0.35	0	3.02	2	10.0	3
15	47.5	4	29.5	4	29.7	4	3.11	2	3.74	3	<0.35	0	1.62	2	2.85	2
16	22.5	4	3.34	2	11.1	3	<0.35	0	1.06	2	<0.35	0	0.63	1	2.56	2
17	73.8	5	12.8	3	69.8	5	33.4	4	38.2	4	<0.35	0	<0.35	0	2.45	2
18	25.6	4	6.60	3	12.0	3	3.76	3	5.91	3	<0.35	0	<0.35	0	<0.35	0
19	17.2	3	5.35	3	18.5	4	1.71	2	2.16	2	<0.35	0	0.40	1	1.77	2
20	37.5	4	9.78	3	37.0	4	6.18	3	6.76	3	<0.35	0	1.03	2	3.27	2
21	31.0	4	9.42	3	28.6	4	5.38	3	5.98	3	<0.35	0	0.85	2	3.36	2
22	35.4	4	11.6	3	21.7	4	<0.35	0	1.01	2	<0.35	0	<0.35	0	5.36	3
23	24.5	4	3.36	2	25.5	4	4.08	3	4.73	3	<0.35	0	0.36	1	4.47	3
24	24.2	4	7.78	3	27.3	4	1.83	2	3.23	2	<0.35	0	<0.35	0	1.82	2
25	77.7	5	6.97	3	15.2	3	6.58	3	7.93	3	<0.35	0	<0.35	0	1.28	2
26	25.4	4	8.16	3	20.9	4	1.58	2	2.00	2	<0.35	0	<0.35	0	2.81	2
27	17.8	4	9.9	3	14.1	3	<0.35	0	<0.35	0	<0.35	0	0.49	1	3.08	2
28	35.4	4	17.8	4	23.4	4	3.51	3	0.39	1	<0.35	0	2.09	2	11.3	3
29	37.1	4	8.82	3	4.99	3	4.99	3	4.57	3	<0.35	0	<0.35	0	<0.35	0
30	41.6	4	4.03	3	34.1	4	16.7	3	16.7	3	<0.35	0	<0.35	0	<0.35	0
31	36.4	4	2.93	2	12.7	3	0.61	1	1.91	2	<0.35	0	<0.35	0	<0.35	0
32	92.4	5	14.2	3	33.7	4	4.21	3	7.11	3	<0.35	0	1.47	2	16.1	3
33	19.0	4	2.16	2	15.9	3	0.35	1	1.62	2	<0.35	0	<0.35	0	<0.35	0
34	44.2	4	4.3	3	18.3	4	0.39	1	<0.35	0	<0.35	0	0.93	2	2.95	2
35	>100	6	58.2	5	98.5	5	15.9	3	20.5	4	<0.35	0	<0.35	0	4.65	3
36	78.3	5	11.5	3	34.5	4	1.89	2	0.89	2	0.46	1	<0.35	0	5.58	3
37	17.1	3	11.4	3	18.1	4	5.74	3	4.73	3	<0.35	0	1.23	2	4.01	3
38	17.2	3	5.56	3	12.4	3	4.22	3	5.16	3	<0.35	0	<0.35	0	0.94	2
39	94.2	5	17.1	3	30.9	4	9.13	3	12.1	3	0.46	1	0.74	2	1.19	2
40	19.0	4	9.86	3	13.0	3	3.02	2	4.03	3	<0.35	0	<0.35	0	<0.35	0
41	6.9	3	1.27	2	6.61	3	<0.35	0	<0.35	0	<0.35	0	<0.35	0	0.63	1
42	51.8	5	13.8	3	22.2	4	0.67	1	0.77	2	<0.35	0	1.15	2	6.09	3
43	14.2	3	4.36	3	15.9	3	5.82	3	6.51	3	<0.35	0	<0.35	0	<0.35	0
44	18.6	4	1.87	2	6.84	3	<0.35	0	<0.35	0	<0.35	0	<0.35	0	<0.35	0
45	4.01	3	2.18	2	3.59	3	<0.35	0	<0.35	0	<0.35	0	<0.35	0	<035	0
46	3.51	3	<0,35	0	1.22	2	<0.35	0	<0.35	0	<0.35	0	<0.35	0	<0.35	0
47	16.1	3	3.00	2	9.48	3	1.91	2	4.85	3	<0.35	0	3.80	3	5.88	3
48	32.2	4	8.73	3	14.0	3	1.43	2	2.40	2	<0.35	0	<0.35	0	0.72	2
49	17.4	3	2.99	2	7.05	3	2.72	2	2.23	2	<0.35	0	<0.35	0	1.64	2
50	21.2	4	15.4	3	23.3	4	<0.35	0	<0.35	0	<0.35	0	<0.35	0	<0.35	0
51	28.8	4	5.82	3	13.8	3	0.89	2	1.01	2	<0.35	0	<0.35	0	0.40	1
52	18.4	4	5.69	3	9.34	3	0.48	1	0.56	1	<0.35	0	<0.35	0	<0.35	0
53	19.0	4	2.02	2	8.52	3	3.58	3	3.81	3	<0.35	0	0.40	1	1.92	2
54	0.90	2	<0.35	0	0.58	1	<0.35	0	<0.35	0	<0.35	0	<0.35	0	<0.35	0
55	13.8	3	7.73	3	10.0	3	0.49	1	2.28	2	<0.35	0	0.49	1	2.39	2
56	5.47	3	1.49	2	4.04	3	<0.35	0	<0.35	0	<0.35	0	<0.35	0	<0.35	0
57	19.0	4	10.6	3	16.0	3	0.84	2	0.58	1	<0.35	0	1.55	2	4.3	3
58	0.69	1	0.35	1	<0.35	0	<0.35	0	<0.35	0	<0.35	0	<0.35	0	<0.35	0
59	52.3	5	14.8	3	34.9	4	6.61	3	9.04	3	<0.35	0	<0.35	0	1.49	2
60	1.58	2	0.41	1	1.27	2	<0.35	0	<0.35	0	<0.35	0	<0.35	0	<0.35	0
61	77.8	5	23.5	4	57.3	5	12.6	3	11.5	3	<0.35	0	4.52	3	9.2	3
62	50.8	5	13.3	3	27.6	4	0.4	1	0.94	2	<0.35	0	<0.35	0	<0.35	0
63	17.0	3	4.73	3	8.27	3	<0.35	0	<0.35	0	<0.35	0	<0.35	0	0.60	1
64	8.46	3	2.28	2	4.69	3	0.54	1	<0.35	0	<0.35	0	<0.35	0	<0.35	0
65	2.80	2	0.40	1	1.45	2	0.35	1	<0.35	0	<0.35	0	<0.35	0	<0.35	0
66	6.85	3	2.56	2	6.23	3	0.52	1	1.09	1	<0.35	0	<0.35	0	0.41	1
67	33.1	4	5.37	3	18.8	4	0.45	1	1.02	2	<0.35	0	<0.35	0	1.6	2
68	31.5	4	8.82	3	15.7	3	0.41	1	0.68	1	<0,35	0	1.02	2	4.87	3
69	3.03	2	<0.35	0	2.61	2	<0.35	0	<0.35	0	<0.35	0	<0.35	0	<0.35	0
70*	67	-	n.d.	-	33	-	<0.3	-	<0.3	-	n.d.	-	n.d.	-	n.d.	-
71**	<0.35	0	<0.35	0	<0.35	0	<0.35	0	<0.35	0	<0.35	0	<0.35	0	<0.35	0

*sIgE determined by ISAC. **Non-allergic control. Sera 52–69 were published elsewhere [40].

Summary of specific Immunoglobulin E against recombinant Bet v 1 and Δ29NCS variants.

### Δ29NCS_5x presents an IgE epitope that cross-reacts with a subset of Bet v 1-homologous allergens

**Figure 3:**
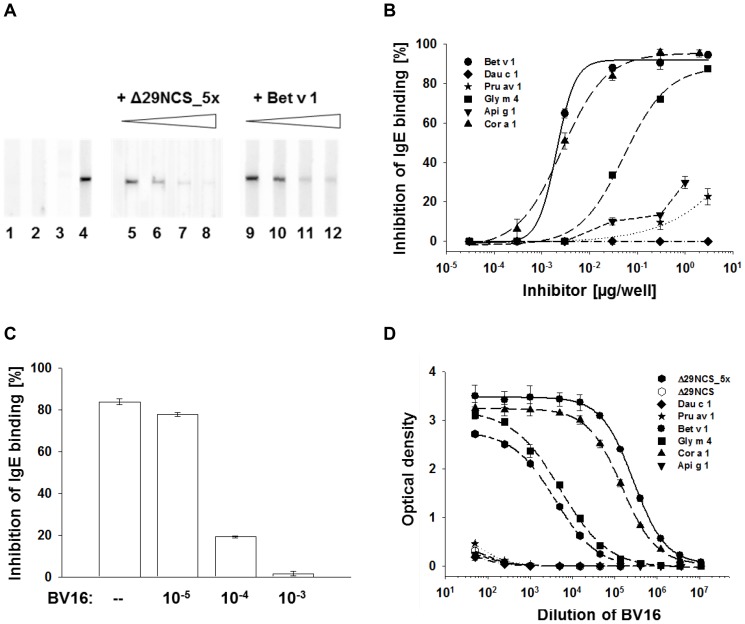
Δ29NCS_5x presents a cross-reactive Ig epitope. (A) Inhibition of IgE binding to Δ29NCS_5x. Binding of serum IgE to Δ29NCS (lane 3) and to Δ29NCS_5x (lane 4) in the presence of increasing amounts of inhibitors Δ29NCS_5x (lanes 5–8) or Bet v 1 (lanes 9–12). No serum (lane 1) and negative serum pool (lane 2) served as control. (B) Dose-dependent inhibition of IgE binding to surface-coated Δ29NCS_5x in the presence of increasing concentrations of PR-10 allergens in ELISA. (C) Inhibition of IgE binding to surface-coated Δ29NCS_5x in the presence of both Bet v 1 and serial dilutions of Bet v 1-binding monoclonal mouse IgG antibody BV16 (–: no BV16; 10^−5^ to 10^−3^: dilutions of BV16) in ELISA. (D) Binding of mouse monoclonal IgG antibody BV16 to surface-coated Bet v 1-related proteins. 250 ng of protein (50 ng of Api g 1) was coated and incubated with dilution series of the monoclonal BV16 in ELISA.

Since the majority of the patients' sera IgE bound Δ29NCS_5x, we asked whether this interaction was due to a Bet v 1-specific IgE epitope presented by the Δ29NCS variant. Therefore we used serum with sIgE to both Bet v 1 and Δ29NCS_5x and tested its IgE interaction with the Δ29NCS_5x variant in the presence of Bet v 1 (Figure 3). As expected, no IgE interaction of the serum pool with Δ29NCS was observed (Figure 3A). In contrast, IgE binding to Δ29NCS___5x was inhibited by Δ29NCS___5x itself and Bet v 1 in a dose-dependent manner, suggesting the presence of an IgE epitope shared by the two proteins.

Next, we asked whether the IgE epitope presented by Δ29NCS_5x is cross-reactive with PR-10 food allergens. Thus, we performed competitive IgE binding experiments (ELISA) with the PR-10 food allergens Cor a 1.04, Gly m 4, Dau c 1.01, Api g 1.01, and Pru av 1, respectively (Figure 3B). Bet v 1 as well as Cor a 1.04 and Gly m 4 competed for serum IgE binding to Δ29NCS_5x, whereas only very low inhibition of IgE binding to Δ29NCS_5x was observed with Api g 1, Pru av 1, and Dau c 1, respectively. We conclude that the IgE epitope offered by Δ29NCS_5x is not ubiquitous but specific for a subgroup of PR-10 allergens.

### The IgE cross-reactive epitope of Δ29NCS_5x overlaps with an IgG binding site

Δ29NCS harbors 47% and Δ29NCS_5x 76% of the structurally resolved IgG epitope (total of 16 amino acids) of Bet v 1, as defined earlier [20]. We therefore asked whether the IgE binding epitope of Δ29NCS_5x overlaps with the binding site of BV16 on Bet v 1. Thus, we tested serum IgE binding of immobilized Δ29NCS_5x in the presence of both Bet v 1 and increasing amounts of BV16 (Figure 3C). In the absence of BV16, IgE binding to Δ29NCS_5x was inhibited to 84% by Bet v 1. The observed Bet v 1-induced inhibition of IgE-Δ29NCS_5x interaction could, however, been abolished by the monoclonal antibody BV16 in a dose-dependent manner. This result suggests a structural overlap between the individual cross-reactive IgE epitope of Δ29NCS_5x and the Bet v 1-specific IgG epitope recognized by monoclonal antibody BV16. Furthermore, when we tested BV16 binding only Bet v 1, Gly m 4, Cor a 1.04, and Δ29NCS_5x interacted markedly with the monoclonal antibody, whereas Dau c 1.01, Api g 1.01 1, Pru av 1, and Δ29NCS did not (Figure 3D).

### Subtle structural differences define the IgE cross-reactivity pattern of Bet v 1-homologous allergens

**Figure 4:**
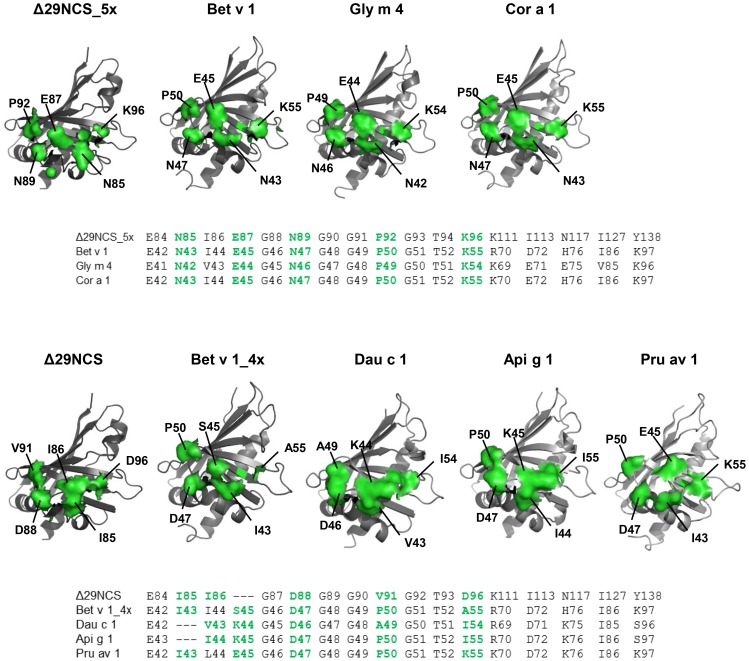
Structural alignment of the Bet v 1 epitope forBV16 and amino acids critical for Ig cross-reactivity in PR-10 allergens. Upper panel: model structures of Δ29NCS_5x and PR-10 allergens showing IgE cross-reactivity with Δ29NCS_5x. The amino acids comprising the BV16-binding epitope and lysine 55 of Bet v 1 and the corresponding residues of Δ29NCS_5x, Gly m 4, and Cor a 1 are listed. Residues of the epitope and of position 55 of Bet v 1 that are critical for Ig cross-reaction are highlighted in green. Lower panel: model structures of Δ29NCS and allergens that do not show IgE cross-reactivity with Δ29NCS_5x. The amino acids corresponding to the epitope in the cross-reactive proteins are listed and highlighted in green.

**Figure 5:**
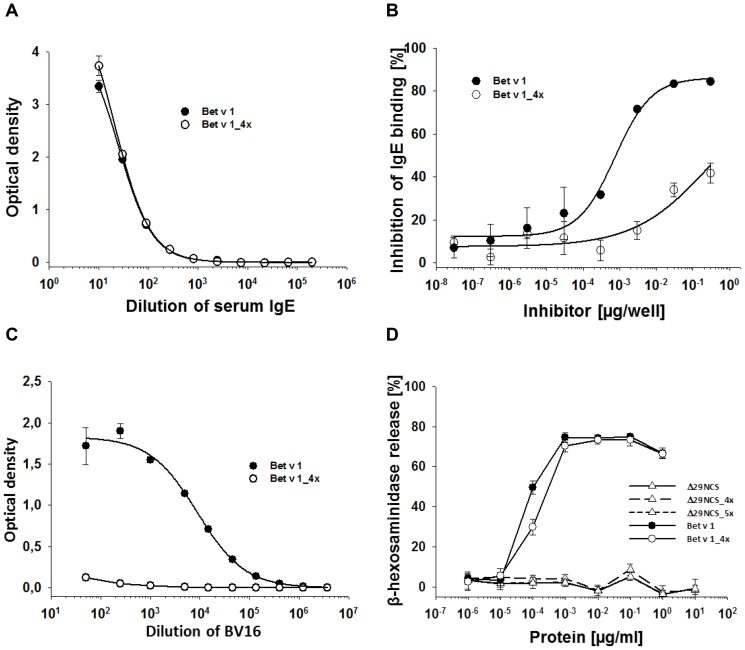
Amino acids critical forIgE and IgG cross-reactivity in a subgroup of Bet v 1-related proteins. (A) Binding of serial dilutions of pool serum IgE to surface-coated equimolar amounts of Bet v 1 and Bet v 1_4x (Bet v 1_N43I/E45S/N47D/K55A_) in ELISA. (B) Inhibition of IgE binding to surface-coated Δ29NCS_5x in the presence of either Bet v 1 or Bet v 1_4x in ELISA. (C) Binding of serial dilutions of monoclonal BV16 to surface-coated equimolar amounts of Bet v 1 and Bet v 1_4x in ELISA. (D) Mediator release induced by recombinant NCS and Bet v 1 variants. Humanized RBL cells were sensitized with a pool of human birch-specific sera. Cross-linking of membrane-bound human IgE by IgE-protein interaction and subsequent release of β-hexosaminidase was determined with serial dilutions of the proteins.

The differential interaction of Δ29NCS_5x and Pr-10 allergens as above with both serum IgE and the Bet v 1-specific mouse monoclonal BV16 let us to hypothesize that Δ29NCS_5x displays an epitope shared only with Bet v 1, Cor a 1.04, and Gly m 4, but not with Dau c 1.01, Api g 1.01, and Pru av 1. Thus, we structurally aligned the epitope grafted onto Δ29NCS_5x and the corresponding surface areas of cross-reactive and non-cross-reactive Bet v 1-related proteins (Figure 4). Residue by residue comparison of the epitope revealed that N43, E45, N47, and K55 of Bet v 1 are shared with Δ29NCS_5x, Gly m 4, and Cor a 1.04, but not with Pru av 1, Api g 1.01, and Dau c 1.01, thus mirroring the IgE/IgG inhibition results (cf. Figure 3) and suggesting that they are critical determinants of IgE cross-reactivity and BV16 binding. To challenge this hypothesis we investigated the substitutional variant Bet v 1_4x (Bet v 1_N43I/E45S/N47D/K55A_). As for Δ29NCS_5x and Δ29NCS_4x, the amino acid substitutions did not alter the protein conformation according to CD and ^1^H^15^N-HSQC NMR spectroscopy (Figure S2). Serum IgE binding to Bet v 1_4x was virtually identical to that of wild type Bet v 1 (Figure 5A). However, inhibition of IgE binding to Δ29NCS_5x was strongly reduced by Bet v 1_4x as compared to Bet v 1 (Figure 5B), and interaction of monoclonal BV16 with Bet v 1_4x was virtually abolished (Figure 5C). The capacity of Bet v 1_4x to induce mediator release in humanized rat basophil leukemia cells *via* allergen mediated cross-linking of receptor bound human IgE was only slightly reduced as compared to wild type Bet v 1, suggesting the presence of further IgE epitopes compensating for the loss of one IgE epitope in Bet v 1_4x (Figure 5D). As expected, the Δ29NCS variants did not induce mediator release due to the lack of a second IgE epitope needed for allergen-mediated IgE cross-linking. Since Bet v 1_4x could not compete with Δ29NCS_5x for binding to serum IgE, we conclude that we have identified an epitope in a subset of PR-10 allergens that binds cross-reactive IgE and IgG.

## Discussion

To analyze epitopes of allergens it is common to genetically engineer specific allergen variants and test their antibody binding capacity in comparison to the respective wild type allergen. A residual response, however, can be misleading if the epitope to be analyzed is conformational and proper binding of the antibody is dependent on the native fold of the allergen. In this case any observed compromised antibody-allergen interaction may well be due to an altered protein conformation of the allergen variant rather than due to the exchange of amino acids crucial for direct antibody binding. Furthermore, the analysis of epitopes in allergens is often hampered by the fact that the polyclonal serum antibody repertoire makes it technically challenging to measure and assign the functionality of single epitopes in the background of the overall multiple antibody binding to the allergen. As the majority of epitopes of inhalative allergens is thought to be conformational and the IgE repertoire of allergic patients to a specific allergen is polyclonal, we circumvented these issues by employing a model protein that i) does not interact with patients' IgE, ii) has a stable allergen-like protein fold, and iii) allows the analysis of individual antibody binding by amino acid substitutions.

Here we analyzed a non IgE-binding variant of the PR-10 enzyme Δ29NCS of the common meadow rue with Bet v 1-type protein structure [25]. We showed that amino acid insertion/substitution did not alter the Bet v 1-like protein fold of Δ29NCS, although it induced significant binding of IgE from sera of birch pollen allergic subjects. IgE that bound to the epitope presented by the model protein Δ29NCS_5x, cross-reacted with a subgroup of PR-10 allergens. As analyzed with a Bet v 1 variant with exchanged residues that mediate antibody binding to the epitope, the observed IgE cross-reaction depended on a subset of amino acids not ubiquitously present in PR-10 allergens. The functional IgE epitope identified in this study overlaps with the known binding site of the Bet v 1-specific murine monoclonal antibody BV16.

The native protein conformation of Bet v 1 and highly likely other PR-10 allergens is critical for IgE interaction since i) neither sequential IgE binding peptides of Bet v 1 nor IgE binding truncated variants of Bet v 1 have been described so far [42] ii) a folding variant of Bet v 1 with native primary structure, but permanently altered secondary structure has very low IgE reactivity [43]. iii) several Bet v 1 variants with amino acid substitutions that induced an altered protein conformation showed reduced binding of serum IgE [7], [44]. Thus we thoroughly studied the secondary and tertiary structure of our model protein by CD and ^1^H^15^N-HSQC NMR spectroscopy. The CD spectra of the Δ29NCS variants were virtually identical and typical for PR-10 proteins. Furthermore, the ^1^H^15^N-HSQC spectra of the Δ29NCS variants were superimposable for the majority of resonances, and the spectra are in agreement with the notion of only minor local differences caused by the amino acid substitutions (cf. Figure 2C), but otherwise identical protein conformation of the Δ29NCS variants. Hence, the Δ29NCS variants have the typical PR-10 protein structure and fulfill the essential requirement for molecular epitope analysis.

When we tested the impact of specific amino acids on serum IgE binding, we found that amino acid substitutions in Δ29NCS with structurally corresponding residues of Bet v 1 induced a significant increase in IgE interaction of the model protein to up to four CAP classes (cf. Table 1). As the protein folds of the Δ29NCS variants were essentially identical, we concluded that the observed serological IgE interaction of the model protein was caused solely by the residues substituted. This hypothesis was further confirmed by the allergen variant Bet v 1_N43I/E45S/N47D/K55A_ (Bet v 1_4x) which showed only little competition for serum IgE binding to Δ29NCS_5x and did not bind the Bet v 1-reactive murine monoclonal antibody BV16. Interestingly, residue E45 of Bet v 1 has already been identified as being critical for both IgE and BV16 interaction [17], [22]. Thus we confirmed the existence of a functional overlapping Bet v 1 epitope for human IgE and murine IgG, with the majority (71%) of our study population having serum IgE against the epitope displayed by Δ29NCS_5x.

Since Bet v 1-specific IgE cross-reacts with PR-10 food allergens [2], we analyzed whether the functional epitope presented by Δ29NCS_5x is also relevant for IgE cross-reactivity. Binding of patients' IgE to Δ29NCS_5x could be inhibited by Bet v 1, Gly m 4, and Cor a 1.04, respectively, suggesting that Δ29NCS_5x displays an epitope shared by these allergens. Interestingly, the PR-10 allergens Pru av 1, Dau c 1.01, and Api g 1.01 did not cross-react with the NCS variant. Since the observed IgE reactivity of the PR-10 allergens we studied correlated well with their binding to the monoclonal antibody BV16, we assumed subtle structural differences within the epitope between the allergens. A sequential/structural alignment of the BV16 epitope indeed revealed that five amino acids are identical and oriented in a similar fashion among the cross-reactive proteins, but not in the non-cross-reactive proteins. To confirm our hypothesis, we generated a corresponding Bet v 1 variant to address these sequential differences and found that the protein variant behaved like the non-cross-reactive Bet v 1-homologous allergens with regard to binding IgG (BV16) and competing with serum IgE for Δ29NCS_5x.

Whereas the structural epitope of an antigen usually spans a molecular surface area of about 400–1000 Å^2^ and comprises 10–25 amino acids, only a small subset of these residues dominates the energetics of the antibody-antigen interaction and thus constitutes the functional (active) epitope [45], [46]. Our model system is ideal for the analysis of functional epitopes, since even low-affinity and individual antibody binding might be detected due to the lack of multiple antibody binding sites in our model protein.

Some information on functional IgE epitopes of Bet v 1 and other PR-10 allergens has emerged in recent years. These studies analyzed the interaction of IgE with either native Bet v 1 isoforms or recombinant allergen variants [8], [9], [12]–[16], [22], [47]. Even though great care had been taken to ensure a Bet v 1-like conformation of the studied protein variants, they were inevitably targeted by a polyclonal IgE antibody response, making it difficult to define the importance and architecture of any single epitope structure. Moreover, bioinformatic approaches are emerging that use either peptide:IgE interaction or structure-based comparison of allergen surfaces to localize Ig epitopes [18], [26], [48]–[50]. We believe that our approach to induce measurable antibody interaction of allergen-like protein variants is ideal to identify individual residues critical for antibody interaction. However, the truncated version of NCS used in this study still carries N- and C-terminal extensions that might locally interfere with epitope analysis. Thus our future research aims to further optimize our model protein system and adapt it to the average number of amino acids of PR-10 allergens.

No mediator release in humanized rat basophil leukemia cells sensitized with sera of birch pollen allergic subjects has been observed with the Δ29NCS variants, indicating that no additional IgE epitope that would mediate IgE receptor cross-linking is displayed in the variants (cf. Figure 5D). In contrast, Bet v 1_4x induced full wild type-like mediator release indicating that the lack of one particular epitope is easily compensated by polyclonal serum IgE. Thus our allergen model system also allows the investigation of the role of epitopes of different affinities to IgE antibodies in the IgE-mediated activation of basophils and mast cells, a crucial step of Type I allergic reactions.

In summary, we have established a protein model system which enables the specific analysis of functional IgE and IgG epitopes and their homologies among PR-10 allergens. Upon single amino acid substitutions of the non-allergenic protein variant Δ29NCS, individual epitopes are identified in a residue-specific manner by detection of newly generated immunoglobulin interactions of the model protein. Our system allows the evaluation of patient-specific epitope profiles and will facilitate both the identification of clinically relevant epitopes as biomarkers and the monitoring of therapeutic outcomes to improve diagnosis, prognosis, and therapy of allergies caused by PR-10 proteins.

## Supporting Information

Figure S1 **^1^H-^15^N-HSQC spectra of different NCS variants.** Spectra of (**A**) 400 µM Δ29NCS (black) (**B**) 250 µM Δ29NCS_N57/I58E/D60N/V63P_ (Δ29NCS_4x) (blue), and (**C**) 400 µM Δ29NCS_N57/I58E/D60N/V63P/D68K_ (Δ29NCS_5x) (red). The proteins were measured in 20 mM sodium phosphate, pH 7.0, 1 mM DTT, 0.04% sodium azide and 10% D_2_O at a 700 MHz spectrometer at 298 K. Due to its solubility limit Δ29NCS_N57/I58E/D60N/V63P_ (Δ29NCS_4x) was measured at a lower protein concentration.
(TIF)

Figure S2 **Folding and structural integrities of Bet v 1 and Bet v 1_4x variant.** (A) Circular dichroism of Bet v 1 and Bet v 1_4x. (B) ^1^H-^15^N-HSQC spectra of Bet v 1 variants. Overlay of the spectra of 100 µM Bet v 1 (petrol) and 100 µM Bet v 1_4x (purple). The proteins were measured in 20 mM sodium phosphate, pH 7.0, 1 mM DTT, 0.04% sodium azide and 10% D_2_O at a 700 MHz spectrometer at 298 K.
(TIF)
