# Supplementary figures and images for: Enlarging the Toolbox for Allergen Epitope Definition with an Allergen-Type Model Protein
Source: PLoS One. 2014 Oct 30;9(10):e111691. doi: 10.1371/journal.pone.0111691 (PMC4214763; doi:10.1371/journal.pone.0111691)

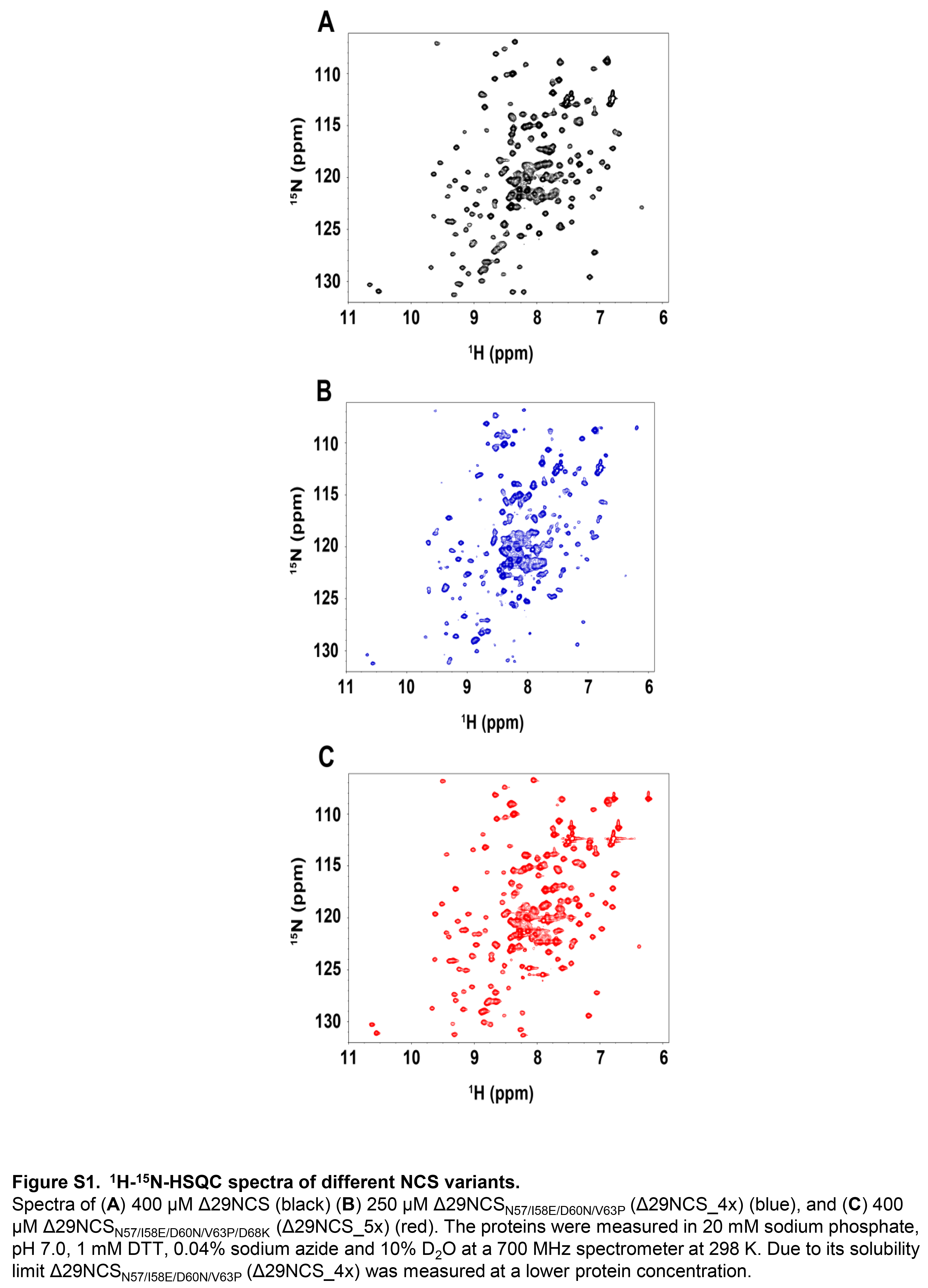

Supplement: Figure S1 — 1H-15N-HSQC spectra of different NCS variants. Spectra of (A) 400 µM Δ29NCS (black) (B) 250 µM Δ29NCSN57/I58E/D60N/V63P (Δ29NCS_4x) (blue), and (C) 400 µM Δ29NCSN57/I58E/D60N/V63P/D68K (Δ29NCS_5x) (red). The proteins were measured in 20 mM sodium phosphate, pH 7.0, 1 mM DTT, 0.04% sodium azide and 10% D2O at a 700 MHz spectrometer at 298 K. Due to its solubility limit Δ29NCSN57/I58E/D60N/V63P (Δ29NCS_4x) was measured at a lower protein concentration. (TIF) [file pone.0111691.s001.tif]

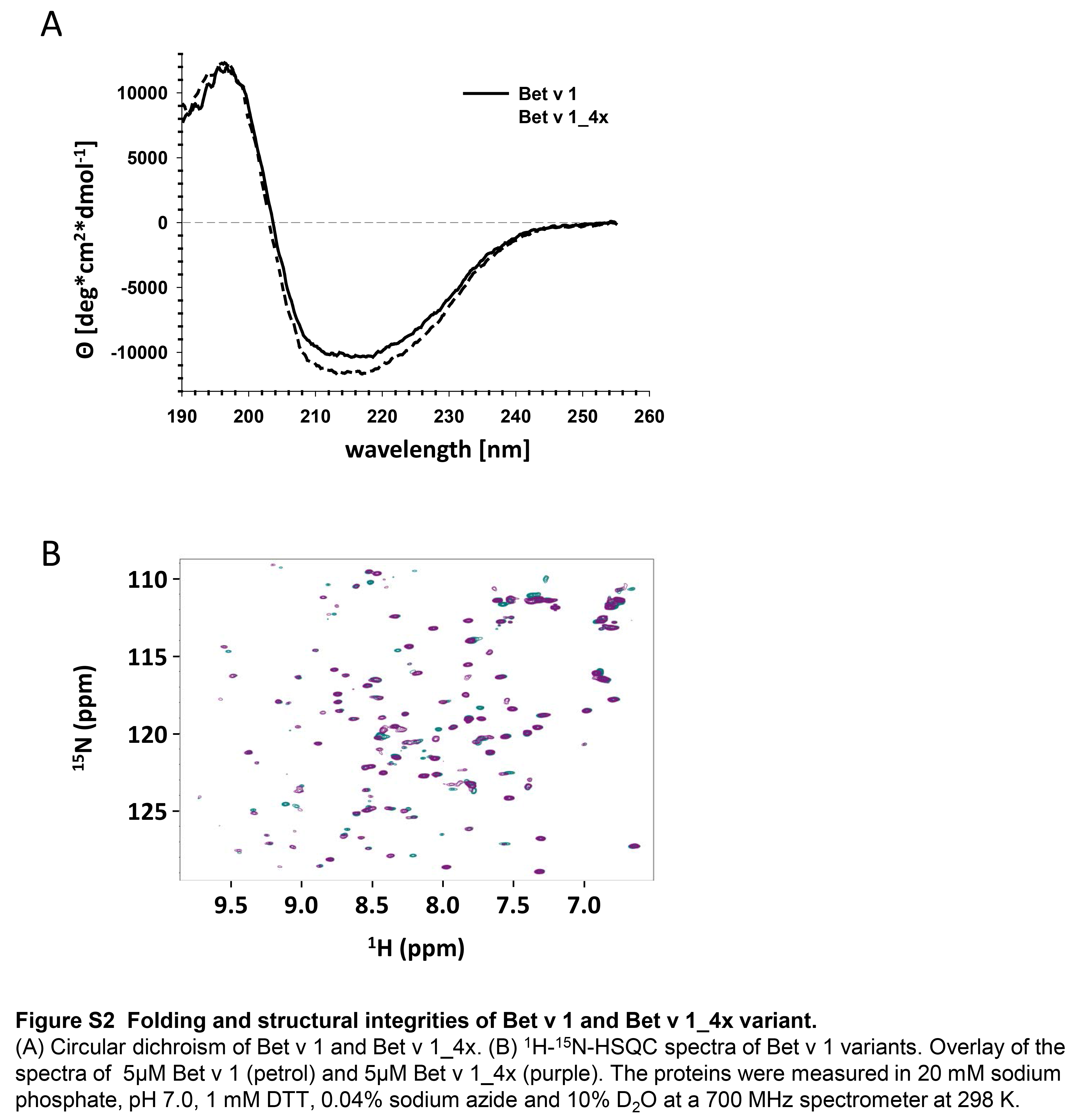

Supplement: Figure S2 — Folding and structural integrities of Bet v 1 and Bet v 1_4x variant. (A) Circular dichroism of Bet v 1 and Bet v 1_4x. (B) 1H-15N-HSQC spectra of Bet v 1 variants. Overlay of the spectra of 100 µM Bet v 1 (petrol) and 100 µM Bet v 1_4x (purple). The proteins were measured in 20 mM sodium phosphate, pH 7.0, 1 mM DTT, 0.04% sodium azide and 10% D2O at a 700 MHz spectrometer at 298 K. (TIF) [file pone.0111691.s002.tif]
